# Effect of Fee on Cervical Cancer Screening Attendance—ScreenFee, a Swedish Population-Based Randomised Trial

**DOI:** 10.1371/journal.pone.0150888

**Published:** 2016-03-17

**Authors:** Emilia Alfonzo, Agneta Andersson Ellström, Szilard Nemes, Björn Strander

**Affiliations:** The Sahlgrenska Academy, University of Gothenburg, Gothenburg, Sweden; Duke University Medical Center, UNITED STATES

## Abstract

**Background:**

Attendance in the cervical cancer screening programme is one of the most important factors to lower the risk of contracting the disease. Attendance rates are often low in areas with low socioeconomic status. Charging a fee for screening might possibly decrease attendance in this population. Screening programme coverage is low in low socio-economic status areas in Gothenburg, Sweden, but has increased slightly after multiple interventions in recent years. For many years, women in the region have paid a fee for screening. We studied the effect of abolishing this fee in a trial emanating from the regular cervical cancer screening programme.

**Method:**

Individually randomised controlled trial. All 3 124 women in three low-resource areas in Gothenburg, due for screening during the study period, were randomised to receive an offer of a free test or the standard invitation stating the regular fee of 100 SEK (≈11 €). The study was conducted during the first six months of 2013. Attendance was defined as a registered Pap smear within 90 days from the date the invitation was sent out.

**Results:**

Attendance did not differ significantly between women who were charged and those offered free screening (RR 0.93; CI 0.85–1.02). No differences were found within the districts or as an effect of age, attendance after the most recent previous invitation or previous experience of smear taking.

**Conclusion:**

Abolishment of a modest screening fee in socially disadvantaged urban districts with low coverage, after previous multiple systematic interventions, does not increase attendance in the short term. Other interventions might be more important for increasing attendance in low socio-economic status areas.

**Trial Registration:**

ClinicalTrials.gov NCT02378324

## Introduction

### Background

Cervical cancer is one of the most common cancers among women globally and in Sweden historically [1]. The disease is considered to be preventable and organised Pap smear screening programmes have decreased incidence and mortality [2]. After screening was introduced in Sweden in 1970, cervical cancer incidence has decreased by 49% and mortality by 72% [3]. Not attending the screening programme is considered to be the most important factor for cervical cancer morbidity in a country with population-based screening [4]. High attendance is crucial for reduction of morbidity and mortality.

Previous studies have identified several factors affecting attendance. The participation rate increased if women were offered scheduled appointments, compared with if they were required to book appointments themselves [5]. Another study showed that if women received a reminder, the attendance rate increased by 9%; if they were reminded by telephone, the rate increased by 31% [6]. A similar conclusion was drawn in a Swedish randomised trial in which increased attendance was observed when midwives contacted non-attendees by telephone [2]. Several randomised trials have found higher attendance when non-attendees were offered HPV self-tests [7–9]. A Swedish study showed that women’s knowledge about the Pap smear’s purpose is low but found no positive association between knowledge and compliance rates [10]. However, other studies have shown a correlation between knowledge about the test and attendance [11, 12]. In a Cochrane review from 2011 concerning cervical cancer screening strategies, the authors found that a formal invitation (e.g. by mail) was associated with a positive trend towards increased attendance and there is some evidence that educational interventions can increase Pap smear uptake [13]. There is also evidence that a standardised invitation was most effective among women who were well informed about the aim of the screening programme or who attended the screening programme regularly, but that more individualised communication is required for less motivated women[14].

Very few studies have investigated the effect of economic incentives or a charged fee on screening participation and the results are conflicting. An English study showed that the attendance rate is influenced by how the unit performing the screening is reimbursed but did not investigate fees for participation [15]. In a meta-analysis regarding interventions that increase attendance in a wide range of screening programmes, it was concluded that offering attendees financial incentives was the second most effective intervention, after organisational interventions [16]. In a small Japanese study, without a control group, providing coupons for free testing was not a generally successful strategy, although it was appreciated among first-time users and was considered to increase awareness of the screening programme [17]. After a policy change in Stockholm, Sweden, a fee was introduced in 2003 and attendance simultaneously dropped by 15% [18].

Denmark, Finland, England and Scotland have no fee for cervical screening while Norwegian women pay the standard fee for a general practitioner visit (≈ €22). In Sweden, most counties charge a fee, ranging from 80 SEK to 200 SEK (≈ 9 €– 22 €), for a Pap smear. In two regions, there is no fee for younger age groups and in two regions there is no screening fee at all [19]. In Region Västra Götaland, where this study was conducted, the fee is 100 SEK (≈ 11 €).

In Sweden, cervical cancer screening programmes are run by the 21 counties. In all counties, Pap smears are taken by midwives at antenatal clinics and scheduled every third year among women aged 23–50 and every fifth year for those aged 50–60. Registers are comprehensive and also include all opportunistic Pap smears outside the organised programmes and data from all laboratories.

Gothenburg is considered to be a highly segregated city with a substantial difference in cervical cancer screening uptake across the city; coverage ranges from 67% to 90%. The populations of the low-resource areas Angered, Bergsjön and Biskopsgården have lower income, lower educational level and poorer health than the region and country in general. Almost 50% of the population in these areas is foreign-born, mostly from countries outside Scandinavia [20]. In order to increase the coverage and attendance rate in Region Västra Götaland, several interventions have been introduced recently: all women receive an invitation for an appointment at a specific time, appointments can be rescheduled online, annual re-invitations are sent to non-attendees until a smear is registered and health care providers can check the date of a woman’s last smear online. During 2011, a specific campaign aiming at increasing attendance was launched in the Angered and Bergsjön areas, including specific interventions such as cultural interpreters and a visiting bus in which drop-in Pap smears were taken free of charge [21]. Although coverage and attendance are low in Gothenburg’s low-resource areas, they are among the districts in the region with the most pronounced increase in attendance in recent years, which can be interpreted as an effect of these systematic interventions [22].

## Aim

Previous studies indicate several possible reasons for lower attendance, but none have studied the importance of a fee in low-income areas. We wanted to study whether abolishment of an existing fee increases attendance in cervical cancer screening in low-resource areas in a setting where several interventions to stimulate attendance had already been implemented.

## Materials and Methods

We conducted a single-blinded, individually randomised controlled trial within the regular cervical cancer-screening programme. The study population consisted of all women in the designated geographical area, consecutively invited for planned screening according to the standard routine. They were thus aged between 23 and 63, with no registered Pap smear during the last three or five years, depending on their age. Re-invitations are sent annually to women who do not attend, as part of the regional screening programme. Study setting: The study took place in three districts in Gothenburg, Sweden, between January 16 and July 12, 2013. Invitations were sent out from January 16 to April 12 and Pap smears taken between February 2 and July 12 were included.

### Interventions

**Fig 1:**
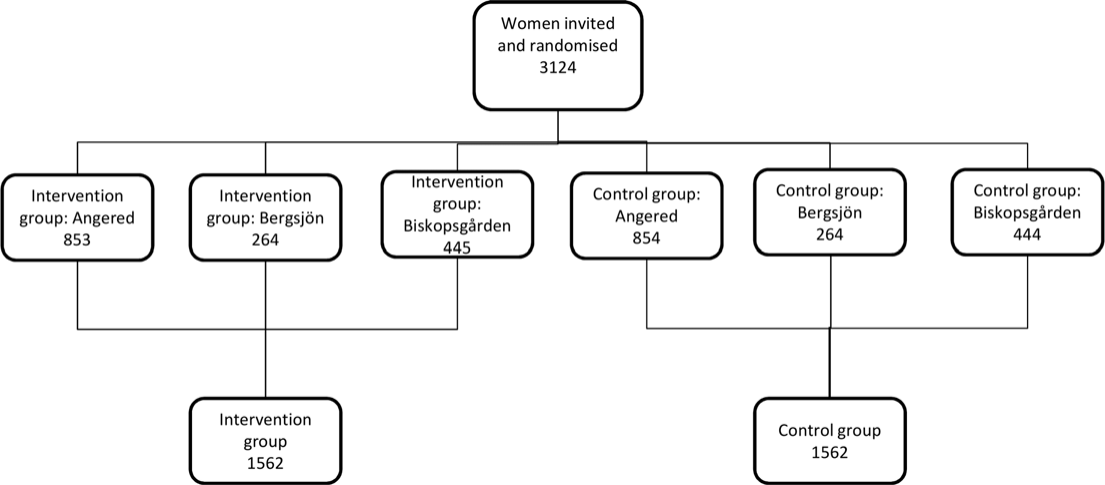
Flow chart of the study. There were no exclusions and no protocol violations.

Women due to be invited for screening were assigned, by individual 1:1 randomisation, to receive an invitation stating either that the test was offered for free (intervention group) or that it cost 100 SEK (control group) (Fig 1). The standard invitation letter was used, offering a scheduled visit at a certain antenatal clinic; the visit could be rescheduled or cancelled online or by phone (S1 File). A short summary in 11 different languages was attached, according to normal routines. Invitation letters were distributed as routine by the Swedish postal service with addresses from the Swedish population register. No study-specific record was kept of letters returned due to incorrect addresses as the population register has high accuracy. During the period 1.3% of all invitations in the county were returned. Attendance data was collected from the Process Registry of the Swedish National Screening Registry, which receives weekly updates on screening activities in western Sweden and includes data on all organised and opportunistic smears.

The primary outcome is difference in attendance. Secondary outcomes are differences in attendance stratified for age groups, home district, previous registered smear and previous non-attendance.

Attendance is defined as a registered Pap smear within 90 days after invitation. All smears are included, regardless of whether they were taken at the designated clinics or elsewhere. Previous smear was defined as one or more smears in the screening registry up to 2012. Previous non-attendance was defined as women who had received an invitation 250–500 days before the present invitation. (Invitations are only sent to women with no registered smears for 3–5 years and are repeated yearly if there is no smear registered. An invitation within the time interval implies that the woman was invited but did not attend).

### Sample size and power calculation

**Fig 2:**
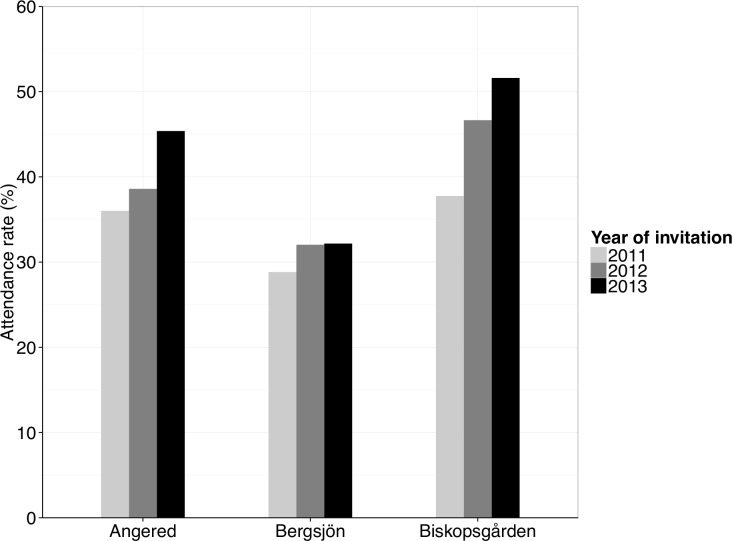
Attendance 2011–2013 in the districts included in the study. Attendance rate is calculated as smears taken within 90 days after sending invitation. Yearly re-invitations to non-participants are not included in the denominators.

Unadjusted attendance in 2010 in Region Västra Götaland was 44%, while it was 23% in Bergsjön, 27% in Angered and 28% in Biskopsgården. The apparently very low rates, particularly in areas with limited coverage, are partly explained by the fact that non-attendees are re-invited every year and thus included in the denominator, which lowers the calculated attendance rate. Attendance, adjusted for re-invitations, is shown in Fig 2. Coverage, calculated as the number of women that have taken a smear during a screening cycle divided by the total number of women in the corresponding age and region, is 86% in the region. The power calculation revealed that 2502 invitations would be needed to detect a relative change in attendance of 20% after the fee was abolished, based on 80% power, a significance level of 0.05 with a two-sided test and 1:1 randomisation. The estimated time needed to recruit enough participants to reach power was three months. In total, 3 124 consecutive women were invited, in order to allow subgroup analysis, thus increasing the power to 88%.

### Randomisation

All women in the catchment areas of Angered, Bergsjön and Biskopsgården that were eligible for invitation in the regular screening programme were individually randomised 1:1 by computer programme into two parallel arms. The intervention group and control group were recorded in the database.

### Blinding

The study was single-blinded. The midwives performing the screening were unaware of whether women had been assigned to the intervention group or the control group. According to existing routines, the control group was billed by mail after the screening.

### Statistical methods

Information from the database regarding the study arm was linked, via unique Swedish personal number, to the Swedish National Cervical Screening Registry, from which aggregated information on attendance was extracted. If a woman had a Pap smear taken at a different clinic from the one at which her smear had originally been scheduled, it was attributed, for the purpose of this study, to the latter. The effect of the intervention on attendance was calculated as relative risks. Confidence intervals for proportions were estimated with the Wilson method. Statistical analyses were performed in R 3.0.2 [23].

### Ethics

In the invitation, the women were informed, according to routine, that data related to screening were registered in a database for quality assurance and that their data could be deleted if they did not consent to this. No women chose this alternative. Therefore no informed consent was obtained, which was approved by the ethical committee. The invitation contained no specific information about the study. Before analysing the results concerning study arm, age group and district, all personal information was anonymised. No information concerning smear results or health status was analysed. The study is registered at ClinicalTrials.gov NCT02378324. Registration was done after inclusion due to human error. No change was made in the protocol after inclusion of patients started. Since the Regional Ethical Review Board at Gothenburg University approved the study Oct 2012 the only changes that were made before the inclusion started in Jan 2013 was to allow the inclusion of 50% more women and to include the neighbouring district Biskopsgården with a similar socioeconomic profile as Angered and Bergsjön. These changes were made to increase power to allow stratified sub-analysis. There was no intermediate control of data during inclusion. The authors confirm that all on going and related trials for this intervention are registered.

### Patient involvement

The research question was raised among women during the previous mentioned public campaign 2011, with several means of communicating with women eligible for screening. Women’s priorities, experiences, preferences and non-attendance in the screening program were agenda setting for developing the study’s design and outcome. The result will not be directly brought back to the women included in the study. Since the study was a part of the regular screening program and aiming at observing differences in behaviour, women/patients did not receive any differential treatment or other active involvement in the study, other than that some women received their testing for free. Thus there was no burden on any participants, and women’s participation/non-participation in the screening did not qualify for any special acknowledgment.

## Results

An overview of the study is shown in Fig 1. A total of 3 124 women from three different districts in Gothenburg were included in the study, i.e. all eligible participants that were consecutively invited, according to the screening programme, from January 16 to April 12, 2013. No protocol deviation was noted. From this population, 1 562 women were randomised to the intervention arm without the screening fee and 1 562 women were randomised to the control arm with the fee.

**Fig 3:**
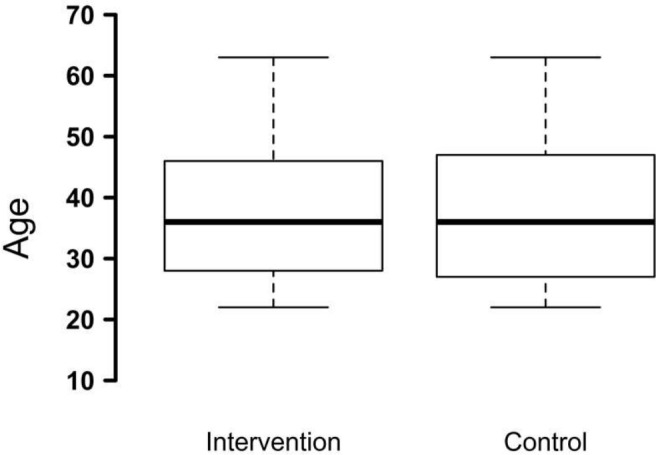
Comparison of age between the intervention and control arm. The central middle line is the median age. The boxes contain 50% of the data, the arms 25 and 25% of the remaining data. No outliers were found.

Randomisation yielded a minor, and statistically non-significant, difference in mean age between the two groups, i.e. 35 vs. 37 years (p = 0,24), although the median age did not differ (Fig 3). There were some minor differences in mean age between the districts. Allocation to the two study arms was evenly distributed within the districts.

**Fig 4:**
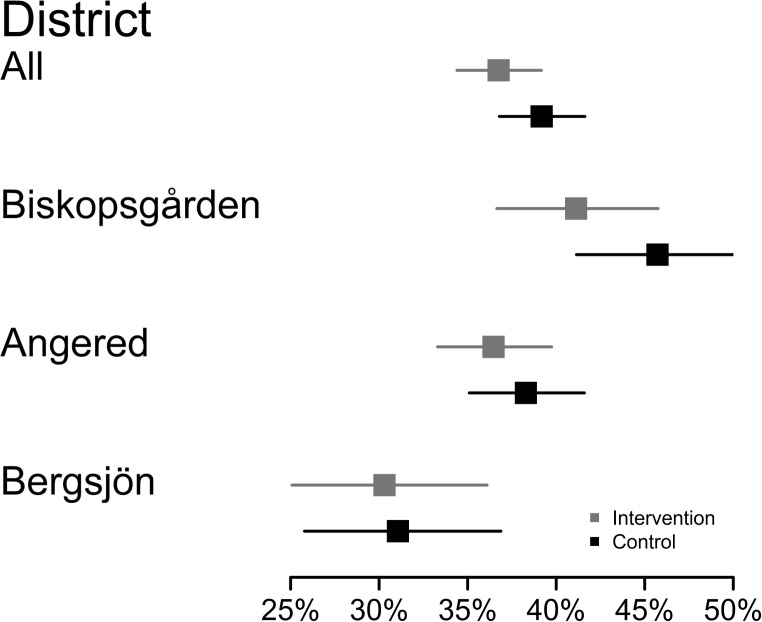
Attendance between the intervention and control group within the districts with 95% confidence intervals.

No significant difference was found between the arms; 36.7% of the women offered free tests participated, compared to 39.2% of those who had to pay (Relative risk 0.93; 95% confidence interval 0.85–1.02) (Table 1). However, the participation rate was significantly lower among the 40–50-year-olds offered free tests (RR 0.72; CI 0.60–0.86). When participation in the districts was compared, we found no significant differences between the study arms in any of the districts (Fig 4). When we stratified for previous Pap smear history (no previous smear compared to one or more previous smears), there were no significant differences between arms within any stratum. (No previous smear: RR 0.87; CI 0.74–1.02; Previous smear: RR 0.98; CI 0.89–1.09). If there was any tendency, it was toward higher participation among those who had to pay, in the group of women without previous smears (Table 2). Among those invited, there were 1 041 women who had not attended after the most recent previous screening invitation. They were equally distributed among the two study arms and the intervention had no effect on their participation (RR 0.88; CI 0.69–1.13) (Table 2).

**Table 1:** Attendance in intervention and control arm.

	Intervention arm—No fee			Control arm—Fee			Relative risk	95% confidence interval
	Invited	Attended		Invited	Attended			
Whole study	1562	574	36.7%	1562	612	39.2%	0.94	0.86–1.03
By area								
Angered	853	311	38.3%	854	327	36.4%	0.96	0.84–1.08
Bergsjön	264	80	30.3%	264	82	31.1%	0.98	0.75–1.26
Biskopsgården	445	183	41.1%	444	203	45.7%	0.90	0.77–1.05
By age								
23–30	536	182	33.9%	545	188	34.5%	0.98	0.83–1.16
31–40	419	170	40.5%	424	165	38.8%	1.05	0.88–1.24
41–50	333	122	36.6%	367	186	50.6%	0.72	0.61–0.86
50 +	274	100	36.5%	226	73	32.3%	1.13	0.88–1.44

Attendance is calculated as smears taken within 90 days after sending invitation. Yearly reminders/re-invitations are included as invitations.

**Table 2:** Attendance in intervention and control arm stratified by previous history of registered smear and previous documented non-attendance.

	Intervention arm—No fee			Control arm—Fee			Relative risk	Confidence Interval
	Invited	Attended		Invited	Attended			
By previous smear								
No previous smear	831	198	23.8%	829	228	27.5%	0.87	0.74–1.02
One or more previous smears	731	377	51.6%	733	384	52.4%	0.98	0.89–1.09
Previous non attendance								
Non attendance	539	98	18.2%	502	103	20.5%	0.89	0.69–1.14

## Discussion

In this randomised study, we found no difference in attendance between women who were offered a free smear and women who had to pay a modest fee. The intervention of abolishing the existing fee had no effect on women´s participation, regardless of whether they had abstained from attending after the most recent previous screening invitation, had a previous smear or not or in which district they lived. Among women aged 41–50 years, participation was higher among those who had to pay.

The study was conducted from January until July 2013. Attendance in both the intervention and control group during the study period was higher than past annual attendance rates in these districts. This is most likely linked to implemented systematic interventions, such as offering rescheduling of screening online, annual re-invitations sent to non-attendees and the possibility for all health providers to see the date of the last smear online, facilitating opportunistic smear-taking. A specific multi-interventional campaign was launched in two of the three districts in 2012. Even one year after the completed campaign and years after introduction of the other interventions, a steady increase in attendance has occurred, as shown in Fig 2.

The study was conducted in districts with low coverage and attendance in the cervical screening programme and with many foreign-born inhabitants. Studies have shown that immigrants from certain regions have a higher incidence of cervical cancer and that attendance among immigrant women is low, especially if they are older [24]. We selected these areas because we assumed that they were districts in which abolishing the fee might have the most profound effect. Thus, we do not know what the effect of abolishing the fee in more affluent areas would have been, but we find it unlikely that attendance would have increased. However, coverage is not optimal in more affluent areas either, and different populations might need different approaches in order to raise attendance.

The abolition of the existing fee was not highlighted in the information, apart from an increase in font size in the text relating to fee in both the intervention arm and the control arm. We chose to handle the information concerning fee/no fee equally, in order to avoid bias. The current fee of 100 SEK (≈ 11€) has been unchanged since 1997. The invitation includes a lot of information and it can thus not be ruled out that some women in the intervention group failed to notice the rephrased sentence stating that the test was offered free. The only changed wording in the information accompanying the invitation concerned the fee. The regular invitation material included a short 50-word summary in different languages that did not contain information about the fee. In the study we kept this unchanged. Non-attendees who might have remembered that a fee was always charged for screening may have missed that key information. However, this is not supported by our findings that there was no difference in participation between the study arms when it came to women without previous registered smears, as well as the youngest women who had not been invited before. The assumption that women abstain due to the fee is also refuted by the finding that a free smear had no impact on attendance among previously non-attending women.

There is evidence that free offers can be regarded as less valuable than if a price is required [25], which might explain the apparent paradox that participation was higher among women aged 41–50 that were required to pay, although a random effect of multiple comparisons should not be ruled out.

The study's strength is the population-based, randomised study design, including all women consecutively invited as part of the regular screening programme in the designated area during three months. The result is therefore based on the entire population eligible for screening during the time interval and targets districts with low attendance in the programme.

There are no other randomised controlled trials addressing the effect of fee on attendance in cervical cancer screening. Our results concur with findings in a meta-analysis, i.e. that organisational interventions were most effective at increasing attendance, while financial incentives were found to be only second most effective [16]. The survey study by Kuroki found an increase in attendance in response to free coupons, but neither the design nor the results allowed any firm conclusions [17].

As previous studies have shown, we may need to focus more on organisational interventions, individualised communication and education to increase attendance in screening, as has been found to be most effective in improving mammography attendance [26].

However, our results are not supported by other findings on breast cancer screening attendance. In a retrospective Finish study, analysing the effect of fee introduction, it was concluded that if women had to pay for mammography, they attended less often [27], regardless of socio-economic status. In a randomised trial from Minnesota, it was concluded that offering mammography screening free of charge increased attendance [28]. One possible explanation for these discrepancies might be that the fee was abolished in our study in addition to other previously implemented measures aimed at increasing participation. Abolishing an existing fee or introducing one might possibly have an effect in settings in which women’s awareness about the positive health effects of screening is low.

In two of the three districts there had been a quite recent campaign to raise attendance. The fact that no difference was observed within any district supports the external validity of our results. However, as this is the first randomised study to investigate the effect of a fee for cervical cancer screening, the results should be interpreted with some caution and more research is needed. It cannot be ruled out that a fee might have a negative effect on attendance in other programmes and settings. The increasing attendance over time in all the three districts indicates an increasing awareness about cervical screening. We cannot rule out that abolishing an existing fee in areas with lower awareness and even lower attendance could have an effect. Our results should not be interpreted as evidence that fees should be introduced in screening programmes that have so far been free of charge, or for raising fees in programmes that charge for participation. Indeed, our results indicate that it might be relevant instead to prioritise other interventions for which there is good evidence that they facilitate screening participation.

## Conclusions

Abolishment of a relatively low screening fee in socially disadvantaged urban districts with low coverage, where systematic interventions to increase attendance in the screening programme had already been implemented, did not increase attendance in the short term. Other interventions might be more important for raising attendance in areas with low socio-economic status.

## Supporting Information

S1 CONSORT Checklist CONSORT Checklist. (DOC)

S1 File Appendix ScreenFee invitation letter. (DOCX)

S1 Protocol Study protocol Swedish. (PDF)

S2 Protocol Study protocol English. (DOCX)

S1 Table Underlying participant data. (XLSX)
