# Supplementary figures and images for: Effect of Fee on Cervical Cancer Screening Attendance—ScreenFee, a Swedish Population-Based Randomised Trial
Source: PLoS One. 2016 Mar 17;11(3):e0150888. doi: 10.1371/journal.pone.0150888 (PMC4795635; doi:10.1371/journal.pone.0150888)

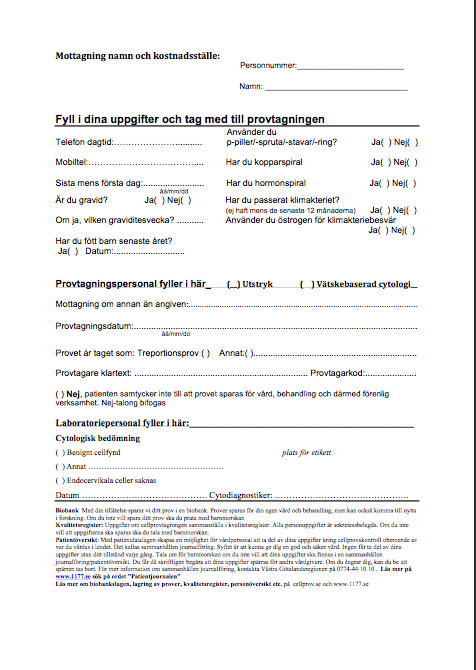

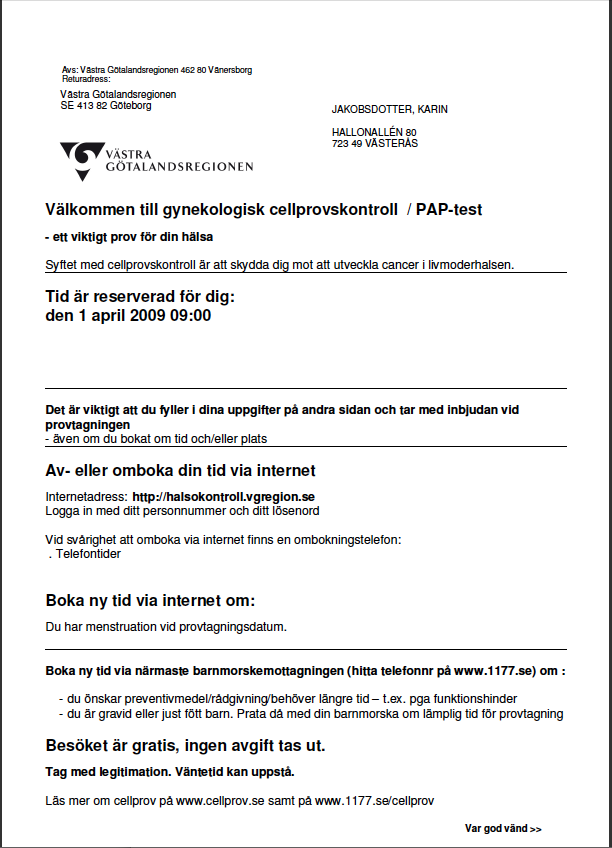

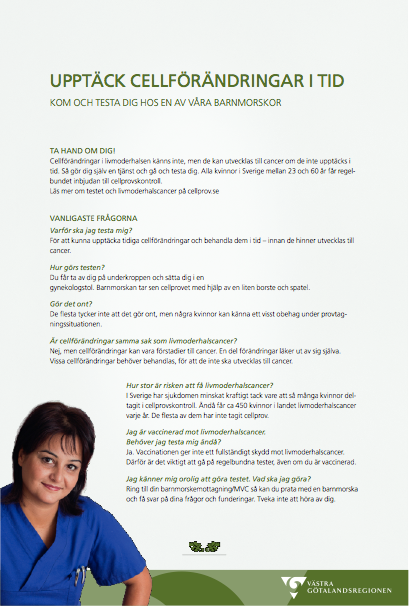

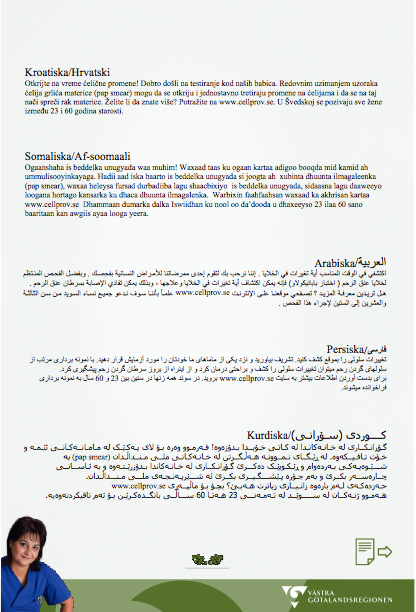


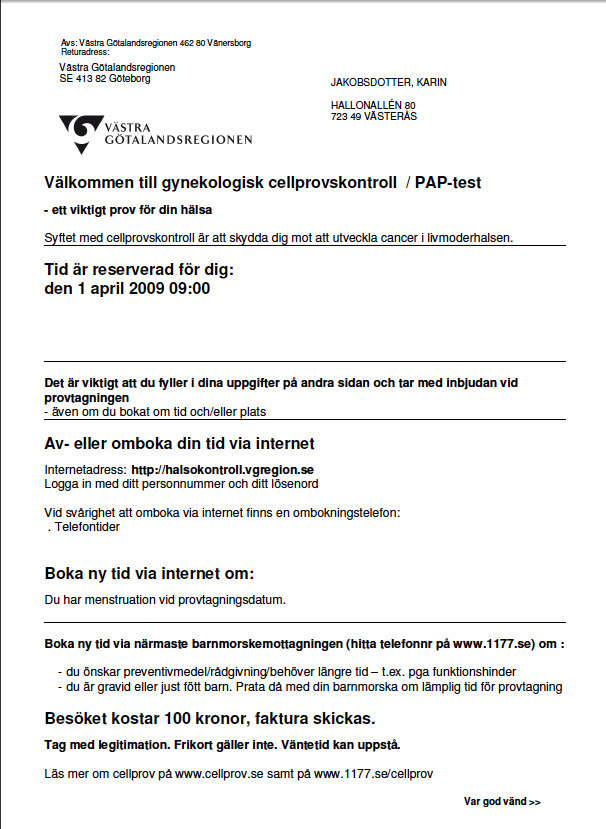

Supplement: S1 File — (DOCX) [file pone.0150888.s002.docx]
