# Supplementary material for: Effect of Fee on Cervical Cancer Screening Attendance—ScreenFee, a Swedish Population-Based Randomised Trial
Source: PLoS One. 2016 Mar 17;11(3):e0150888. doi: 10.1371/journal.pone.0150888 (PMC4795635; doi:10.1371/journal.pone.0150888)
Supplement: S1 Protocol — (PDF) [file pone.0150888.s003.pdf]

EPN Regionala etikprövningsnämnden i Göteborg

Projektansvarig: Björn Strander
SU/Sahlgrenska
Regionalt cancercentrum väst
413 45 Göteborg

Dnr: 742-12

Exp. 2012-10-19

Forskningshuvudman: Västra Götalandsregionen

Närvarande:
Gunnar Dyhre, *ordförande*
Claes Corlin, *bitr. vetenskaplig sekreterare*

Ledamöter med vetenskaplig kompetens:
Dennis Beach, *jävig ärende 677-12*
Elisabeth Björk Brämberg, *jävig ärende 739-12*
Sally Boyd
Anna-Karin Kollind
Lena Lindgren, *jävig ärende 747-12*
Jesper Lundgren
Bibbi Ringsby Jansson

Ledamöter som företräder allmänna intressen:
Erling Alriksson
Lisbeth Ekman
Bengt Fernström
Marianne Henningsson
Pia-Lotta Ranmalm Lagerlöf

Projekttitel: Betydelsen av avgift för deltagande i screening - kvalitetsarbete på vetenskaplig grund
Projekt ID: VGFOUGSB-257961

Beslutsprotokoll från sammanträde med Regionala etikprövningsnämnden i Göteborg, Avdelningen för övrig forskning, den 15 oktober 2012

Föredragande: Anna-Karin Kollind

Godkännes

Nämnden beslöt godkänna studien.

Att denna avskrift i transumt överensstämmer med originalet intygar:

Barbro Morsing, byråsekreterare

Regionala etikprövningsnämnden i Göteborg
Box 401, 405 30 Göteborg
Besöks- och leveransadress: Guldhedsgatan 5A, 413 20 Göteborg
Tel: 031-786 68 21, 786 68 22, 786 68 23, Fax: 031-786 68 18
www.epn.se

ORIGINAL ABH

2012 -09- 25

ABII

ANSÖKAN OM ETIKPRÖVNING

Information till ansökan, ***se bilaga och Vägledningar*** ***(www.epn.se)***

742 - 12

Till Regionala etikprövningsnämnden i: Göteborg

Den regionala etikprövningsnämnd till vars upptagningsområde forskningshuvudmannen hör, se respektive nämnd *(www.epn.se)*

Avgift inbetald datum: ~~120917~~ 120921

Observera att en ansökan aldrig är komplett och därmed kan behandlas förrän blanketten är korrekt ifylld och avgiften är betald.

Projekttitel: Betydelsen av avgift för deltagande i screening - Kvalitetsutvecklingsarbete på vetenskaplig grund.

Ange en beskrivande titel på svenska för lekmän, utan sekretesskyddad information. Ange också i förekommande fall projektets identitet, projektets/forskningsplanens (protokollets eller prövningsplanens) nummer, version, datum osv.

Projektnummer/identitet: VGFOUGSB-257961 Version nummer: 1

Uppgifter som fylls i av den regionala etikprövningsnämnden

Ansökan komplett: 120925	Dnr: 742 - 12
Begäran om ytterligare information (i sak):	Begärd information inkommen:
Beslutsdatum: 121015	Expeditionsdatum: 121019

Ansökan avser (gäller även vid begäran om rådgivande yttrande):

Forskning där endast en forskningshuvudman deltar (5 000 kr)	☐
Forskning där mer än en huvudman deltar (16 000 kr)	☐
Forskning där mer än en forskningshuvudman deltar, men där samtliga forskningspersoner eller forskningsobjekt har ett omedelbart samband med endast en av forskningshuvudmännen (5 000 kr)	☐
Endast behandling av personuppgifter (5 000 kr)	☒
Forskning som gäller klinisk läkemedelsprövning (16 000 kr)	☐
Ändring av tidigare godkänd ansökan enligt 4 § förordning (2003:615) om etikprövning av forskning som avser människor (2 000 kr)	☐

Om nämnden finner att forskningsprojektet inte faller inom etikprövninglagens tillämpningsområde önskas ett rådgivande yttrande. (Info: 4a och 4b §§ i förordning 2003:615) (Info: Bilaga till ansökan)

Ja: ☒ Nej: ☐

1. Information om forskningshuvudman m.m.

1:1 Forskningshuvudman (Info: p. 1:1 i Vägledning till ansökan)

Ansökan om etikprövning av forskning ska göras av forskningshuvudmannen. *Med forskningshuvudman avses en statlig myndighet eller en fysisk eller juridisk person i vars verksamhet forskningen utförs.* Inom staten utförs forskning främst vid lärosätena, men även vid vissa andra myndigheter, som t.ex. Brottsförebyggande rådet och Socialstyrelsen. Kommuner och landsting kan vara forskningshuvudmän, liksom privaträttsliga juridiska personer.

Namn: Västra Götalandregionen

Adress:

1:2 Behörig företrädare för forskningshuvudmannen

Behörig företrädare är t.ex. prefekt, enhetschef, verksamhetschef. Forskningshuvudmännen bestämmer själva, genom interna arbets- och delegationsordningar eller genom fullmakt, vem som är behörig att företräda forskningshuvudmannen. Kopia av sådan handling *ska* bifogas.

Namn: Katerina Storek Tjänstetitel: Verksamhetschef

Adress: Enheten för gynekologi och mödravård, Primärvården Göteborg

Gynekologmottagningen, Majornas Vårdcentral, Skärgårdsgatan 4, 41458 Göteborg

1:3 Forskare som är huvudansvarig för genomförandet av projektet (kontaktperson)

(Info: p. 1:3 i Vägledning till ansökan)

Namn: Björn Strander Tjänstetitel: Med dr, Överläkare

Adress: Regionalt cancercentrum väst

Sahlgrenska Universitetssjukhuset, 413 45 Göteborg

E-postadress: bjorn.strander@oc.gu.se

Telefon:

Mobiltelefon: 0704-972226

1:4 Plats (Info: p. 1:4 i Vägledning till ansökan)

Plats (er) där projektet ska genomföras, ange inrättning (ar), institution (er), klinik (er) etc.

Primärvården, Göteborg

1:5 Andra medverkande

Övriga deltagande forskningshuvudmän samt forskare ansvariga för att lokalt genomföra projektet (kontaktpersoner) anges här eller i bilaga med namn och adresser (se p. 9 bilaga nr 1).

Med dr Agneta Andersson-Ellström, Primärvårdens FoUU-avdelning, Göteborg

1:6 Ansökan/anmälan till andra myndigheter

Vid läkemedelsprövning

Ansökan om tillstånd av *Läkemedelsverket* – se Läkemedelsverkets hemsida (www.mpa.se)

Ansökan inlämnad (datum) Tillstånd erhållits ☐

EudraCT nr:

Vid viss genetisk forskning

Om personuppgifter om genetiska anlag som har framkommit efter genetisk undersökning kommer att hanteras i studien ska detta anmälas till *Datainspektionen* enligt 10 § personuppgiftsförordningen (1998:1191) – se Datainspektionens hemsida www.datainspektionen.se

Anmälan inlämnad (datum) Kommer att inlämnas efter godkänd etikprövning ☐

Vid viss forskning som innefattar bestrålning av forskningspersoner (Info: p. 9 i Vägledning till ansökan)

Ansökan, enligt 16 och 22 §§ Strålsäkerhetsmyndighetens föreskrifter (SSMFS 2008:35) om allmänna skyldigheter vid medicinsk och odontologisk verksamhet med joniserande strålning, till *Strålskyddskommitté* – för vidare information kontakta aktuell lokal strålskyddskommitté.

Ansökan inlämnad (datum): Ansökan tillstyrkt ☐

2. Uppgifter om projektet

2:1 Sammanfattande beskrivning av forskningsprojektet (programmet)

Vägledning till forskningsplan/forskningsprotokoll (program) (Info: p. 9 i Vägledning till ansökan)

Beskrivningen ska kunna förstås av nämndens samtliga ledamöter. Undvik därför terminologi som kräver specialkunskaper. Ange bakgrund och syfte för studien samt den/de vetenskapliga frågeställning (ar) som man söker svar på. Ange de viktigaste undersökningsvariablerna. Beskriv vilka kunskapsvinster projektet kan förväntas ge och betydelsen av dessa. Ange om det är en registerstudie, uppdragsforskning etc. För fackmän avsedd detaljerad information i forskningsplan/forskningsprotokoll (program) *ska* bifogas som bilaga (se p. 9 bilaga nr 2). En utförligare beskrivning av studiens genomförande *avsedd för lekmän* kan vid behov bifogas den för fackmän avsedda obligatoriska forskningsplanen.

Deltagande i gynekologisk cellprovskontroll är avgörande för att slippa få livmoderhalscancer och att undvika att dö i sjukdomen. Frågan om hur deltagande ska kunna förbättras i cancerscreeningprogrammen är en specifik del av regeringens nationella cancerstrategi. Data från kvalitetsregister visar att deltagandet varierar ganska mycket mellan olika delar av regionen och mellan stadsdelarna i Göteborg. Flera projekt har genomförts för att finna metoder att öka deltagandet, främst i socioekonomiskt missgynnade områden. Gynekologisk cellprovskontroll är idag avgiftsbelagd. Internationellt sett är det ovanligt med avgift i screening. Landstingen i Sverige har olika policies. Sannolikt utgör avgiften ett visst hinder för deltagande, men det finns ingen säker kunskap om detta vilket bl.a. SKL har efterlyst. Vi har startat ett projekt för att förbättra och utveckla screeningprogrammet på vetenskaplig grund. Vi vill erbjuda 1100 kvinnor i nordöstra Göteborg, som har det lägsta deltagandet i regionen, gratis provtagning. För att kunna utvärdera effekten görs detta randomiserat så att en kontrollgrupp får betala avgift på sedvanligt sätt. Om deltagandet ökar med 20% anser vi detta vara betydelsefullt och en relevant effekt. I den databas som används för att skicka ut kallelser gör vi en notering om kvinnan tillhör "gratisgruppen" eller gruppen som ska betala sedvanliga 100 kr för provet. 90 dagar efter utskickandet av inbjudan följer vi upp deltagandet i det Regionala kvalitetsregistret för gynekologisk cellprovskontroll "Cytburken" som Regionalt cancercentrum Väst ansvarar för. Resultatet av detta pilotprojekt kommer att ligga till grund för om projektet utvidgas till att omfatta hela regionen. Resultatet kommer att få stor nationell betydelse för frågan om avgift ska förekomma i cancerscreening och möjligen också för andra screeningprogram.

2:2 Vilken/vilka vetenskaplig (a) frågeställning (ar) ligger till grund för projektets utformning?

Om projektet kan karakteriseras som en hypotesprövning, ange den primära och eventuellt sekundära hypotesen. Hänvisning till mer detaljerad information för fackmän kan ske till bifogad forskningsplan enligt punkt 2:1

Hypotesen är att avskaffande av avgiften för gynekologiskt cellrpovskontroll ökar deltagandet med minst 20% i nordöstra Göteborg

2:3 Redogör för resultat från relevanta djurförsök

Om djurförsök inte utförts ange skälen till detta.

ej aktuellt

2:4 Redogör översiktligt för undersökningsprocedur, datainsamling och datas karaktär

(Info: p. 2:4 i Vägledning till ansökan)

Av beskrivningen ska framgå hur projektet planeras genomföras. Beskriv insamlade datas karaktär. Ange hur datas tillförlitlighet säkerställs (t.ex. kvalitetskontroll/monitorering). - Vid enkäter och intervjuer ska beskrivas tillvägagångssätt och t.ex. frågors innehåll och hur slutsatser dras. Enkäter och skattningsskalor *ska* bifogas (se p. 9 bilaga nr 5). - För medicinsk forskning ska anges t.ex. typer av ingrepp, mätmetoder, antal besök, tidsåtgång vid varje försök, doser och administrationssätt för eventuella läkemedel och/eller isotoper, blodprovsmängd (även ackumulerad mängd vid multipla försök). Ange även om och på vilket sätt undersökningsprocedur m.m. skiljer sig från klinisk rutin. Ange proceduren för att ge den eventuella behandling efter projektets slut, som kan erfordras. Ange procedur för insamling av biologiskt material. Redogör för datakällor och procedurer vid behandling av personuppgifter. För mer detaljerad information kan hänvisning ske till bilagd forskningsplan.

2200 konsekutiva kvinnor, aktuella för inbjudan, inkluderas och randomiseras 1:1. I den databas som ligger till grund för utskick av kallelser till gynekologisk cellprovskontroll markeras om inbjudan innehåller erbjudande om kostnadsfri undersökning eller inte.

Uppgiften länkas till regionala databasen för gynekologisk cellprovskontroll (Cytburken). Från denna databas uthämtas aggregerade data om deltagande i respektive arm, räknat som prov inkommet inom 90 dagar efter inbjudan har skickats ut. Länkning av data sker genom personnummer. Endast uppgift om typ av erbjudande och senare deltagande kopplas till personuppgift, inga känsliga personuppgifter som provresultat eller hälsotillstånd. Uppföljning sker med data som i vanlig rutin rapporteras in till befintliga register.

Alla personuppgifter avidentifieras före analys. Relativ risk beräknas liksom chi-två-test för signifikansberäkning.

2:5 Redogör för om insamlat biologiskt material kommer att förvaras i en biobank

(Info: p. 2:5 i Vägledning till ansökan)

Med biobank avses biologiskt material från en eller flera människor som samlas och bevaras tills vidare eller för en bestämd tid och vars ursprung kan härledas till den eller de människor från vilka materialet härrör. Redogör för var och hur prover som ska sparas förvaras, kodningsprocedurer och villkor för utlämnande av prover. Ange huvudman för biobanken. Observera att i förekommande fall ska anmälan av biobank ske till Socialstyrelsen enligt lagen (2002:297) om biobanker i hälso- och sjukvården m.m.

Ej relevant

2:6 Redovisa tillgång till nödvändiga resurser under projektets genomförande

Ange vem/vilka som har ansvaret (prefekt, verksamhetschef eller motsvarande) för forskningspersonernas säkerhet vid alla enheter/kliniker där forskningspersoner ska delta. Intyg från dessa ansvariga *ska* bifogas (se p. 9 bilaga nr 9). Av intyget ska framgå att erforderliga ekonomiska, strukturella och personella resurser finns tillgängliga för att garantera forskningspersonernas säkerhet.

Erforderliga resurser finns. Se bilaga

2:7 Journalföring, registrering och hantering av data (Info: p. 2:7 i Vägledning till ansökan)

Redogör för hur undersökningsprocedurer och eventuella ingrepp journalförs. Ange hur registrering och behandling av resultaten ska gå till. Om materialet ska kodas, ange proceduren, vem som förvarar kodlistor och vem eller vilka som har tillgång till dem, var och hur länge de förvaras samt om materialet kommer att anonymiseras eller förstöras. Ange om band- och videoinspelningar används. Redogör för vilken tillgänglighet datamaterialet har och hur det förvaras samt hur erforderligt sekretesskydd erhålls.

Som framgått av ovanstående sker en markering av vilken typ av inbjudan som går ut i kallelsesystemet. Detta arbetar i normal driftsmiljö hos VGR IT med servicenivå 4 vilket innebär; tillgänglighet under dagtid, 15 minuters informationssäkring, 2 timmars riktvärde för incidenter, max nedtid 8 timmar. Uppgifterna länkas mot Regionala kvalitetsregistret för cervixcancerprevention vid Regionalt cancercentrum väst (Cytburken) som driftas vid ITS, Umeå universitet, likaledes i skyddad, säkrad miljö. Endast statistikern vid RCC kommer att behandla identifierbara uppgifter. Analyserna på gruppnivå görs avidentifierat.

2:8 Redogör för tidigare erfarenheter (egna och/eller andras) av den använda proceduren, tekniken eller behandlingen

Särskilt angeläget är att redovisning av risker för komplikationer görs tydliga och i förekommande fall med angivande av relevanta publikationer. Vid nya behandlingar av patienter, t.ex. med läkemedel, bör anges hur många patienter (med aktuell eller annan åkomma) som tidigare erhållit föreslagen behandling, läkemedelsdosering (eller annan dosering) samt hur långa behandlingsperioder som studerats.

Regionalt cancercentrum Väst som bl.a. inrymmer regionala cancerregistret och ett flertal nationella och regionala kvalitetsregister har omfattande erfarenhet av databehandling av personuppgifter

3. Uppgifter om forskningspersoner

3:1 Hur görs urvalet av forskningspersoner? (Info: p. 3:1 i Vägledning till ansökan)

Med forskningsperson avses en levande människa som forskningen avser. Ange urvalskriterier (inklusion och exklusion). Redogör för på vilket sätt forskaren kommer i kontakt med/får kännedom om lämpliga forskningspersoner. Ange om rekrytering sker från egna/andras tidigare eller pågående studier. Om annonsering sker, *ska* annonsmaterialet insändas som bilaga (se p. 9 bilaga nr 3). Om t.ex. barn eller personer som tillfälligt eller permanent inte är kapabla att ge ett eget informerat samtycke ska ingå i projektet, ska detta särskilt motiveras. Om vissa grupper (t.ex. kvinnor, barn eller äldre) utesluts från deltagande i projektet ska detta särskilt motiveras.

De kvinnor som får erbjudande om gratis undersökning väljs ut slumpvis genom randomiseringsförfarande av 2200 konsekutiva kvinnor som får inbjudan till mödravårdscentralerna i Bergsjön och Angered efter projektets början

3:2 Ange relationen mellan forskare/försöksledare och forskningspersonerna

- ☐ Behandlare (t.ex. läkare, psykolog, sjukgymnast) - forskningsperson (t.ex. patient, klient)
- ☐ Kursgivare (lärare) - student
- ☐ Arbetsgivare - anställd
- ☐ Annan relation som kan tänkas medföra risk för påverkan. Beskriv: Ingen relation

3:3 Redogör för det statistiska underlaget för studiepopulationens (-ernas)/ undersökningsmaterialets (-ens) storlek (Info: p. 3:3 i Vägledning till ansökan)

Redovisa statistisk styrka, så kallad "power"- beräkning eller redovisa motsvarande överväganden som tydliggör studiens möjligheter att besvara frågeställningarna.

Hörsamheten (andel med prov registrerat inom 90 dagar efter att kallelse skickats) är idag för hela VGR 44 %, för Bergsjön 23 % och för Angered 27 %. Powerberäkningen grundar sig på att man via projektet med avgiftsfritt test skulle uppnå en 20% ökning av hörsamheten i Bergsjön och Angered vilket bedöms kliniskt relevant. För att uppnå en power på 80% med en signifikansnivå av 0,05 med enkelsidig test, randomiserade 1:1, behöver man då kalla minst 1972 kvinnor.

3:4 Ange om forskningspersonerna kan komma att inkluderas i flera studier samtidigt eller i annan/andra studie (-er) i nära anslutning till denna? I så fall vilken typ av forskning? (Info: p. 3:4 i Vägledning till ansökan)

Ej aktuellt

3:5 Vilket försäkringsskydd finns för de forskningspersoner som deltar i projektet?

Det åligger forskningshuvudmannen att kontrollera att befintliga försäkringar täcker eventuella skador som kan uppkomma.

Ej relevant

3:6 Vilken ekonomisk ersättning eller andra förmåner utgår till de forskningspersoner som deltar i projektet och när betalas ersättningen ut? Utförligare beskrivning kan lämnas i bilaga.
(Info: p. 3:6 i Vägledning till ansökan)

Ersättning för obehag och besvär. Belopp (före skatt):

Ersättning för förlorad arbetsinkomst	☐ Ja	☒ Nej
Reseersättning	☐ Ja	☒ Nej
Befrielse från kostnader för läkemedel	☐ Ja	☒ Nej

Befrielse från andra kostnader. Vilka? Deltagande i gynekologisk cellprovskontroll

Andra förmåner. Vilka?

När betalas ersättningen ut?

Ingen ersättning betalas ut ☒

4. Information och samtycke (Info: Forskningspersonsinformation)

4:1 Proceduren för och innehållet i den *information* som lämnas då forskningspersoner tillfrågas om deltagande

Beskriv hur och när information ges och vad den innehåller. Ange vem som informerar. Normalt ska en kortfattad och lättförståelig skriftlig information ges. Denna skriftliga information *ska* bifogas ansökan (se p. 9 bilaga nr 4). Om ingen eller ofullständig information ges, måste skälen för detta noggrant anges.

I inbjudan till Gynekologisk cellprovskontroll framgår att personuppgifter kring provtagning behandlas, att uppgifter överförs till kvalitetsregister, att kvinnan kan välja att utplåna uppgifter ur kvalitetsregister samt hur hon ska förfara för detta. Rutiner för detta finns upprättade (men har faktiskt aldrig behövt användas eftersom ingen av de många hundra tusen kvinnor som hittills inbjudits har valt att utträda ur kvalitetsregistret!) Skulle någon kvinna välja att inte vilja ha sina uppgifter i kvalitetsregister kommer hon att exkluderas ur kvalitetsuppföljningen. Någon ytterligare information kommer inte att lämnas eftersom vi inte redovisar specifikt någon av de andra variablerna som vi inhämtar och utvärderar ur kvalitetsregister. Att detta projekt innehåller ett erbjudande som inte ges till alla är i sig inte unikt. Vi har åldersgränser för screeningundersökningar, olika uppföljningar för lätta cellförändringar om man är under eller över 35 år etc och dessa inklusioner och exklusioner utvärderas ständigt. Det som möjligen är speciellt är att detta projektet bättre kan utvärderas vetenskapligt genom att det sker en randomisering.

4:2 Hur och från vem inhämtas *samtycke*?

Beskriv proceduren; vem som frågar, när detta sker och hur samtycket dokumenteras. Utförlig redovisning är särskilt viktig då barn eller personer med nedsatt beslutskompetens ingår i studien, likaså vid studier av en grupp/grupper, t.ex. föreningar, organisationer, företag, kyrkosamfund, församlingar eller skolklasser.

Kvinnan som inbjuds kan välja att avstå från att delta i kvalitetsregister, se ovan

5. Forskningsetiska överväganden

5:1 Redogör för de risker som deltagandet kan medföra samt möjliga komplikationer

Dessa kan vara t.ex. fysisk skada, smärta, obehag eller integritetsintrång som projektet innebär eller kan innebära. Ange vilka åtgärder som har vidtagits för att förebygga de risker som nämns ovan samt vilken beredskap som finns för att hantera sådana komplikationer. Ange vilka/de metoder som kommer att användas för att efterforska, registrera och rapportera oönskade händelser.

Inga risker.

5:2 Redogör för förutsebar nytta för de forskningspersoner som ingår i projektet

De som får erbjudande om gratis provtagning spar pengar och kan möjligen ta ett prov av bevisat medicinskt värde som man inte skulle tagit annars

5:3 Gör en egen värdering av förhållandet risk - nytta för de forskningspersoner som deltar

Tveklöst enbart nytta

5:4 Identifiera och precisera om etiska problem t.ex risk - nytta i ett vidare perspektiv kan uppstå inom eller genom projektet

Här kan redovisas om exempelvis vissa grupper kan komma att utpekas/få hjälp som ett resultat av studien.

Inga kvinnor som får ett erbjudande om gratis provtagning kan förutses ha några som helst men av detta, inte heller att effekten av erbjudande utvärderas i kvalitetsregister. Samma gäller för dem som slumpas till konrollgrupp. Kvinnorna som får gratis provtagning har en direkt nytta av detta, medan de som utgör kontrollgrupp får oförändrade förhållanden .

6. Redovisning av resultaten

6:1 Hur garanteras forskningshuvudmannen och medverkande forskare tillgång till data (anges vid t.ex. uppdragsforskning) och vem ansvarar för databearbetning och rapportskrivning?

(Info: p. 6:1 i Vägledning till ansökan)

6:2 Hur kommer resultaten att göras offentligt tillgängliga? Kommer studien att insändas för publicering i tidskrift eller publiceras på annat sätt?

Ange i vilken form resultaten planeras offentliggöras samt tidsplan för detta.

Resultatet kommer att inkluderas i den kunskapsbas som kommer att ligga till grund för det nya regionala vårdprogram för cervixcancerscreening som börjat tas fram. (Även ett nationellt program finns planerat men ännu ej beslutat) Det kommer att redovisas för regionala styrgruppen för cervixcancerprevention och Hälso och sjukvårdsutskottet i VGR. Vidare inför projektledningen för deltagande i screening inom SKL och Regionala cancercentras samordnings Nationella arbetsgrupp för cervixcancerprevention. Resultatet kommer att presenteras vid den nationella konferens om deltagande i cervixcancerscreening som planeras 2013. Eftersom nuvarande policyn att ta betalt vid screeningundersökningar är mycket ovanlig internationellt, är det tveksamt om resultatet kan publiceras i en internationell tidskrift, men bör kunna publiceras i Läkartidningen.

Resultatet kommer med all sannolikhet att få stor nationell betydelse för organiserad screeningverksamhet fr.a. mot livmoderhalscancer men troligen också för andra screeningprogram (mammografi, bukaorta m fl.)

6:3 På vilket sätt garanteras forskningspersonernas rätt till integritet när materialet offentliggörs/publiceras?

Redovisas resultat på statistisk gruppnivå? Beskriv procedurer eller metoder för avidentifiering/anonymisering.

Bearbetning och analys kommer att ske avidentifierat. Vi kan inte se några integritetsproblem i sammanhanget.

7. Redovisning av ekonomiska förhållanden och beroendeförhållanden

Redovisning enligt punkterna 7:1-7:3 syftar till att tydliggöra alla direkta eller indirekta förhållanden, som kan tänkas påverka forskarens relation till forskningspersonerna (vid t.ex. informations-, samtyckes-, genomförandeprocedurer).

7:1 Vid uppdragsforskning

Ange uppdragsgivaren t.ex. ett företag (vid klinisk läkemedelsprövning eller prövning av andra nya produkter), en organisation eller en myndighet.

Namn: Kontaktperson:

Adress: Telefon/mobiltelefon:

Ange uppdragsgivarens relation till forskningshuvudmannen/medverkande forskare, t.ex. anställningsförhållande

7:2 Redovisa eventuella ekonomiska överenskommelser med uppdragsgivare eller andra finansiärer (namn, belopp)

Vid klinisk läkemedelsprövning bör hänvisning ske till ingånget avtal med sjukvårdshuvudmannen. Liknande överenskommelser kan förekomma vid annan uppdragsforskning och ska redovisas på samma sätt. Separata överenskommelser med den/de som ska genomföra forskningen ska redovisas. Belopp som kommer att erhållas för studien/ersättning till kliniken/genomföraren, vad ersättningen ska täcka och ev. belopp som erhålls per forskningsperson, ska också anges här (se p. 9 bilaga nr 12).

Ej relevant. Projektet finansieras av FoUU-avdelningen, Primärvården Göteborg

7:3 Redovisa forskningshuvudmannens, huvudansvarig forskares och medverkande forskares egna intressen

Här redovisas t.ex. aktieinnehav, anställning, konsultuppdrag i finansierande företag, eget företag som kan få (direkt eller indirekt) ekonomisk vinst av forskningen (se p. 9 bilaga nr 12).

Ej relevant

8. Undertecknande

Behörig företrädare för sökande forskningshuvudman enligt p. 1:2

Ort: Göteborg Datum: 12-09-22

Signatur:

Namnförtydligande: Katerina Storek

Tjänstetitel: Verksamhetschef

Undertecknad forskare som genomför projektet (kontaktperson) enligt p. 1:3 intygar härmed att forskningen kommer att genomföras i enlighet med ansökan

Ort: Göteborg Datum: 120922

Signatur: Björn Strander

Namnförtydligande: Björn Strander

Tjänstetitel: Överläkare

9. Förteckning över bilagor (Info: p. 9 i Vägledning till ansökan)

Dokument som, i tillämpliga fall, ska bifogas *om inte motsvarande information finns i blanketten* har markerats med x. Markera de bilagor som skickas in med denna ansökan.

Insänd med ansökan	Bil nr	Beskrivning	Klinisk läkemedels-prövning	Annan forskning
☐	1	Deltagande forskningshuvudmän och medverkande forskare (kontaktpersoner) vid forskning där mer än en forskningshuvudman deltar. Info p. 1:5	x	x
☐	2	För fackmän avsedd forskningsplan, vid behov även för lekmän avsedd bilaga. Info p. 2:1 och i Vägledning till forskningsplan/forskningsprotokoll (program)	x	x
☐	3	Annonsmaterial för rekrytering av forskningspersoner. Info p. 3:1 och i Vägledning till ansökan p. 3:1	x	x
☒	4	Skriftlig information till dem som tillfrågas. Info p. 4:1 och i Forskningspersonsinformation	x	x
☐	5	Enkät, frågeformulär. Info p. 2:4	x	x
☐	6	Gemensam EU blankett (gäller fr.o.m. den 1 maj 2004), gäller även vid ändring.	x	
☐	7	Sammanfattning av protokollet på svenska	x	
☐	8	Prövarhandbok alt. bipacksedel/produktresumé/IB	x	
☒	9	Intyg från verksamhetschef/motsv. om resurser och om forskningspersonernas säkerhet. Info p. 2:6	x	x
☒	10	CV för forskare (samma som p. 1:3) med huvudansvar för genomförandet, redovisa forskarens (-arnas) kompetens av relevans för studien. Info i Vägledning till ansökan p. 1:3	x	x
☐	11	Beskrivning av ersättning till forskningspersoner. Info p. 3:6 och i Vägledning till ansökan p. 3:6	x	x
☐	12	Överenskommelser med uppdragsgivare/finansiär om t.ex. anställningsförhållanden, bidrag/ersättning till prövningsplats, sjukvårdshuvudman, forskningshuvudman eller forskare. Info p. 7:2 och p. 7:3	x	x

Övriga bilagor som bifogas ansökan:
