# Supplementary material for: Effect of Fee on Cervical Cancer Screening Attendance—ScreenFee, a Swedish Population-Based Randomised Trial
Source: PLoS One. 2016 Mar 17;11(3):e0150888. doi: 10.1371/journal.pone.0150888 (PMC4795635; doi:10.1371/journal.pone.0150888)
Supplement: S2 Protocol — (DOCX) [file pone.0150888.s004.docx]

**Regional Ethical Review Board at Gothenburg University**

**Responsible for the study**:  **Dnr**: 742-12 **Exp**: 2012-10-19

Björn Strander

Sahlgrenska University hospital

Regional cancer centre west

41345 Göteborg

**Principal scientific authority**: Region Västra Götaland

Attending:

Gunnar Dyhre, president

Cales Corlin, ass. Scientific secretary

**Members with scientific competence**:

Dennis Beach

Elisabet Björk Brämberg

Sally Boyd

Anna-Karin Kollind

Lena Lindgren

Jesper Lundgren

Bibbi Ringsby Jansson

**Members representing the public’s interest:**

Erling Alriksson

Lisbeth Ekman

Bengt Fernström

Marianne Henningsson

Pia-Lotta Ranmalm Lagerlöf

**Project title:** The importance of fee in attendance in cervical cancer screening- quality work on scientific basis.

Project ID: VGFOUGSB-257961

Decision protocol from the regional Ethical Review Board at Gothenburg University October 15^th^ 2012

The committee decided to approve the study.

**Application for ethics approval**

Decision by ethical committee: 121015

1. 1. **Principal scientific authority where the research will be performed:**

Region Västra Götaland.

- 1. **Representative of the scientific authority:** Katarina Storek. Operational chief Dep och gynecology and antenatal care, Primary health care Göteborg. Gynecological clinic, Majorna HC, Skärgårdsgatan 4, 41458 Göteborg.
  2. **Researcher in charge of the study:** Björn Strander, Consultant, Regional Cancer centre west. Göteborg.
  3. **Place**: Primary health care in Göteborg
  4. **Others participating:** Agneta Andersson-Ellström, Jene Winge.

**2. Information about the project**

**2.1 Description of the research project:** Attendance in cervical cancer screening is crucial to avoid cervical cancer and avoid mortality in the disease. How to increase attendance in cervical cancer screening program is a specific part of the government’s national strategy against cancer. Data from quality registers show that attendance varies a lot between different parts of the region and between different districts in Göteborg. Many projects have been performed, aiming at find strategies to increase attendance, mostly in socioeconomically disadvantaged areas. Today a fee is charged on Pap-smears. Of international point of view it is rare with fees within screening. The counties in Sweden have different policies. It is likely that a fee might be an obstacle for attendance, but there is no certain knowledge about this, which for example SKL (Swedish communes and counties) have asked for. We have started a project to improve and develop the screening program on scientific basis. We want to offer 1100 women in north eastern Gothenburg, which has the lowest attendance in the region, testing for free. To evaluate the effect, this will be done in a randomized way and the control group will pay the fee as usual. If attendance increases by 20% we consider it as important and a relevant effect. In the data base that we use for calling patients for screening we will note if the women belongs to the fee-free group or the usual group that pay 100 SEK for the test. 90 days after invitation is sent to participants we will follow up attendance in the regional quality register for gynecological pap smear testing “Cytburken” (Process Registry of the Swedish National Screening Registry) that Regional cancer center west is responsible for. The result of this pilot project will be the basis if the project will extend to include the whole region. The result will have great national importance for the question if fees will occur in cancer screening and possibly also for other screening programs.

- 1. **Which scientific question is the background of the project?**

The hypothesis that abolishment of fee for cervical cancer screening increase attendance with at least 20% in northeastern Gothenburg.

- 1. **Animal testing:** Not applicable
  2. **Data collection:**

2200 consecutive women, due to screening, are included and randomized 1:1. In the database that handles the regular screening invitations the invitation is marked with normal fee or without fee.

Information is linked to the Process Registry of the Swedish National Screening Registry, which receives weekly updates on screening activities in western Sweden and includes data on all organised and opportunistic smears. From this database aggregated data about attendance in each group is retrieved, counted as a registered Pap smear within 90 days after invitation. Data is linked via personal number. Only information about type of offer and attendance is linked to personal number, no sensitive personal information as test result and health status. Follow up is done with data that as usual is reported to existing register.

All personal data is anonymized before analysis. Relative risk is counted as well as chi-two test for significance calculation.

- 1. **Will biological material be stored in a bio banc?** Not applicable.
  2. **Describe necessary resources during the project:** Sufficient resources are available.
  3. **Data collection, data registration and handling of data:**

As described above there will be a note in the database of which kind of invitation is sent to the participants. The database is within Region Västra Götaland IT with service level 4, which implicates: availability daytime, 15min of information security, 2hours of benchmark for incidents, maximum time down 8hours. Information is linked to Process Registry of the Swedish National Screening Registry, which is operated at ITS, Umeå University also in a safe and protected environment. Only the statistician at Regional Cancer centre west will handle information that can be linked to personal numbers. The analysis at group level will be performed with anonymised data.

- 1. **Describe previous experience about the procedure, technique or treatment:**

Regional Cancer centre west also includes the regional cancer register and multiple national and regional process registers and has wide experience of handling data of personal information.

1. **Information about participants**

3.1 How is the selection of participants made?

The women receiving a fee free offer are randomly assigned by randomisation of 2200 consecutive women receiving invitation in Bergsjön and Angered after the project’s start.

- 1. **Relation between researcher and participants:** No relation.

**3.3** **Describe for the statistical background of the study population:**

Attendance (registered Pap smear within 90 days after invitation) is today in Region Västra Götaland 44%, in Bergsjön 23% and in Angered 27%. Power calculation is based upon data that via the project and fee free test will reach a 20% increased attendance in Bergjön and Angered which is a clinical relevant effect. To reach an 80% power with a significance level of 0. 05 with one sided test, randomised 1:1, at least 1972 women need to be invited.

- 1. **State if the participants will be included in several studies at the same time or in another study in close relation to this one?** Not applicable.

**3.5 Which insurance covers the participants?**Not applicable.

**3.6 Which economic incentive is given to participants?** Not applicable.

**4. Information and consent**

**4.1 The procedure for and the content in the information that is given to participants due for participation:**

In the invitation, the women were informed, according to routine, that data related to screening were registered in a database for quality assurance and that their data could be deleted if they did not consent to this. There are routines for this (but has never been used, because none of all hundreds of thousands of women have choose to be withdrawn from the Process register). If any woman chooses not to have her data in the Process registry she will be excluded. Any further information will not be provided because we do not present any other variable that we can get through the Process register. This project contains an offer that is not given to everyone, is unique. We have age limits for screening investigations, different follow up for LSIL if the woman is under or above 35 years etc. and these inclusions and exclusions are constantly evaluated. This project can better be evaluated scientifically if it is performed with randomisation.

**4.2 From whom and how is consent given?** The women that are invited can choose to refrain from participating in Process registry. Se above.

**5. Ethical considerations:**

**5.1 Describe the risks participation can lead to:** No risks.

**5.2 Describe any possible advantage for participants:**

The ones getting a free test save money and can possibly take a test of a medical value that wouldn’t have been taken otherwise.

**5.3 Valuate the relation risk and gain for participants:** Only gain.

**5.4 Identify and precise if ethical problem e.g. risks, gain in a wider perspective:**

No woman getting an offer of a free fee can be anticipated getting any associated risks, neither that the effect of offer will be evaluated in a Process register. The same for those in the control group. Women receiving the free fee have directly advantage, the women in the control group have unchanged conditions.

**6. Presentation of the results**

**6.2 How will the results be publically available? Will the study be published?**

The results will be included in a database that will be the basis for the new regional care program for cervical cancer screening (also a national program is planned but no decision is yet taken). It will be presented for the Regional board for cervical cancer screening and the Health care committee in Region Västra Götaland. It will also be presented for the project management for attendance in screening within SKL (Swedish communes and counties) and the National Working Group for Cervical Cancer Prevention. The result will be presented at the national conference about attendance in cervical cancer screening that is planned 2013. Because the present policy to take a fee for screening investigations is very rare internationally, it is uncertain if the results will be published in an international magazine, but will be published in Läkartidningen (Swedish doctor’s magazine). The results will probably get great national importance for organized screening above all in cervical cancer screening but probably also for other screening programs (mammography, abdominal aortic aneurysm).

**6.3 How is the integrity of the participants guaranteed when the study is published?**

Data analysis will be done with anonymized data. No integrity problems will occur therefore.

**7. Describe economic relations**

**7.1 For research by commission:** Not applicable

**7.2 Describe possible economical agreements with commissioners or other financial groups:** Not relevant. The project is financed by a grant from Research and Development in Närhälsan Primary Care, Region Västra Götaland.

**7.3 Describe the researchers own interest:** Not applicable.

**Attachment to the application**

1. Written information for the participants.
2. Certificate from operational chief regarding funding of the project, that enough economic resources are available and the security of the participants in the project.
3. CV of the researchers.
